# Incorporating equity, diversity, and inclusiveness into the Hong Kong Principles

**DOI:** 10.1371/journal.pbio.3001140

**Published:** 2021-04-27

**Authors:** David Moher, Lex Bouter, Nicole Foeger, Ulrich Dirnagl

**Affiliations:** 1 Centre for Journalology, Clinical Epidemiology Program, Ottawa Hospital Research Institute, Ottawa, Canada; 2 School of Epidemiology and Public Health, University of Ottawa, Ottawa, Canada; 3 Department of Epidemiology and Biostatistics, Amsterdam University Medical Centers, Amsterdam, the Netherlands; 4 Department of Philosophy, Faculty of Humanities, Vrije Universiteit, Amsterdam, the Netherlands; 5 Austrian Agency for Research Integrity, Vienna, Austria; 6 Berlin Institute of Health, QUEST Center for Transforming Biomedical Research, Berlin, Germany

## Abstract

In this response to Labib and Evans, authors of the Hong Kong Principles look forward to collaborating with those from the broad research integrity community to ensure that issues of equity, diversity and inclusion will become part of the ecosystem of research integrity.

We thank Ms. Labib and Dr. Evans for their work on the Hong Kong Principles (HKPs) [1]. Your letter raises important points and gives us an opportunity to respond and clarify our perspective. As you indicate in your letter, equity, diversity, and inclusion (EDI) are important topics that require attention in the assessment of researchers. We did not address them specifically in the HKPs because our main focus was on rewarding responsible research practices that improve the transparency and validity of research. As a clarification (and not to be interpreted as defensive on our part), EDI also wasn’t mentioned prominently in our breakout discussions with more than 100 participants of the 6th World Conference on Research Integrity, where the draft HKPs were discussed and finalized.

In our paper [2], Principle 5 (recognize essential other tasks like peer review and mentoring) provides a useful example that illustrates that the essence of the Labib and Evans comments about EDI are in alignment with our views—“Macquarie University, Sydney, Australia, has some exciting initiatives in their new academic promotion policy, which includes five pillars, one of which is in leadership and citizenship. Here, researchers can show their alignment with the university’s values and broader contribution to the university and its community [87]. Since this policy was introduced, it has been reported that the number of promotion applications increased by 50%, and the number of women promoted has also increased [88]”.

We think there will be an opportunity to more forcefully address EDI in the envisioned Cape Town Statement on Fostering Research Integrity through equity, fairness, and diversity (programmed as a focus track on the 7th World Conference on Research Integrity, Cape Town, South Africa, 2022). We can imagine that the Cape Town Statement will demand fostering EDI in research, including in the assessment of researchers for hiring, promotion, or tenure. We would see this as an update of the HKPs.

We are pleased that the HKPs has initiated discussion on EDI as part of the process of assessing researchers for hiring, promotion, and tenure. We look forward to collaborating with Labib and Evans and many others from the broad research integrity community to ensure that EDI will become part of the ecosystem of research integrity.
